# Governing accelerated evidence in real-time clinical trials

**DOI:** 10.1038/s43856-026-01909-8

**Published:** 2026-10-28

**Authors:** Azizi A. Seixas

**Affiliations:** 1 Department of Informatics and Health Data Science, University of Miami Miller School of Medicine, Miami, FL USA; 2 Department of Psychiatry and Behavioral Sciences, University of Miami Miller School of Medicine, Miami, FL USA

**Keywords:** Adaptive clinical trial, Randomized controlled trials

## Abstract

The FDA’s real-time clinical trial initiative marks a pivotal shift in regulating clinical trials, yet accelerating data flows may outpace the governance, statistical safeguards, and inclusive infrastructure required for trustworthy evidence. To address these issues, the commentary identifies four key challenges and proposes a three-pillar governance framework to ensure rigorous, equitable, and continuously adaptive oversight and operations.

## Introduction

**Fig. 1:**
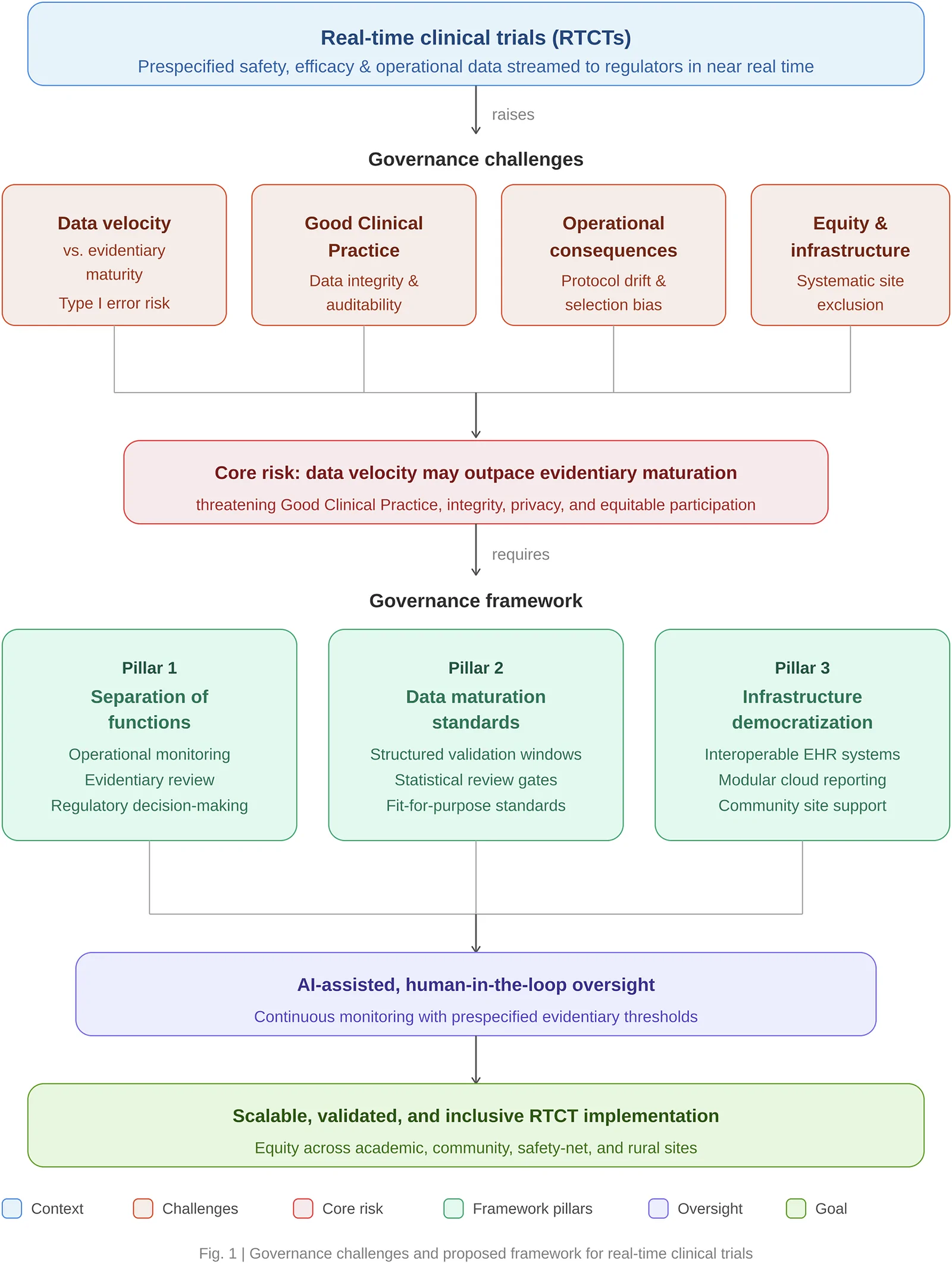
Governance challenges and proposed framework for real-time clinical trials. RTCTs raise four interrelated challenges that threaten the integrity of regulatory-grade evidence --- (i) evidentiary maturity, (ii) Good Clinical Practice, (iii) operational and behavioral consequences, and (iv) inequitable infrastructure access that threaten regulatory-grade evidence. Left unaddressed, these challenges risk undermining the research epistemological, operational, ethical and translational foundations that clinical trials depend on.   A three-pillar governance framework is proposed to address them directly, built on   separation of operational monitoring, evidentiary review, and regulatory decision-making; structured data maturation and statistical review standards; and infrastructure democratization for community, safety-net, federally qualified health center, and rural health systems. Underpinning all three pillars, AI-assisted, human-in-the-loop oversight ensures that  scalable, validated, and equitable RTCT implementation remains continuously governed rather than passively monitored.

The FDA’s recent initiative to implement real-time clinical trials (RTCTs), with AstraZeneca’s Phase 2 TRAVERSE and Amgen’s Phase 1b STREAM-SCLC trials where real-time safety, efficacy, and operational data are transmitted to regulators, marks a consequential shift in clinical evidence^1^. Although RTCTs promise to modernize clinical development, the central concern is whether accelerating data velocity will outpace the evidentiary maturation, independent oversight, and infrastructure investment required to protect Good Clinical Practice, data integrity, and equitable participation across the full spectrum of trial sites (Fig. 1). These concerns are neither hypothetical nor premature. Live data are already flowing to the FDA from two operational trials, and the governance and infrastructure challenges examined here are preconditions for any trustworthy scaling of the program^2^. The best time to establish governance is now, before RTCTs scale beyond conditions that current program infrastructure was designed to handle.

## Issue 1: Data velocity, accelerated evidence, and evidentiary maturity

Clinical trials provide the highest level of evidentiary strength in medicine with prespecified analyses, bias mitigation, endpoint adjudication, disciplined analysis, maturation of data via validation, and protection against random errors. RTCTs promise transparency, operational efficiency, and strengthened safety monitoring, but each advantage carries a corresponding risk. Regulatory visibility may influence sponsor behavior, continuous data review may function as unscheduled interim analyses, and real-time monitoring may drive premature interpretation of immature signals, increasing the risk of multiplicity, Type I error, evidentiary lifecycle compression, and unstable and nonreproducible data, raising concerns that near–real-time data transmission may expose research information before it has sufficiently matured for interpretation. Addressing these risks does not require slowing the pace of innovation; rather, it requires purpose-built governance for real-time environments that establish when data are mature enough to interpret, who is authorized to act on them, and what evidentiary standards must be met before regulatory conclusions are drawn. Without this governance, RTCTs risk swinging the pendulum too far, trading the inefficiencies of episodic evidence generation (a system that already fails more than 90% of Phase I candidates before they reach approval) for something potentially more damaging, a real-time evidence ecosystem that questions the veracity, integrity, and maturity of trial data, undermining the very foundation that makes clinical trials the highest level of evidence in medicine^3^.

## Issue 2: Good clinical practice

Good Clinical Practice represents the universally accepted ethical and scientific standards of clinical trial data to ensure their credibility, accuracy, and protection of participants, foundational pillars upon which regulatory decisions and public trust in clinical evidence rest. RTCTs place that foundation under direct pressure. As trials increasingly rely on electronic health records, wearable devices, and AI-enabled data pipelines, near-real-time data capture compresses the verification, adjudication, and oversight timelines that GCP depends on, while demanding simultaneous compliance with validation, auditability, and access control standards under 21 CFR Part 11^4,5^. Continuous data ingestion and regulatory transmission create concrete risks to data provenance, privacy and security concerns, version control, and reproducibility, particularly in decentralized settings where data originate across heterogeneous systems with variable quality. The FDA’s real-world evidence framework requires that data used for regulatory decision-making be fit for purpose, yet in RTCT environments the critical question is not only whether data meets that standard but when a distinction exists that existing GCP frameworks were not designed to operationalize under continuous data flow^6^. Preserving GCP therefore requires governance that defines fitness for purpose not as a fixed threshold but as a continuously governed and auditable process.

## Issue 3: Operational and behavioral consequences

Beyond technical and statistical considerations, RTCTs introduce operational and behavioral uncertainties that may affect trial conduct and internal validity. Continuous data visibility may increase observation of emerging signals, yet current RTCT proposals provide limited clarity regarding how such data will be interpreted or acted upon in practice^7^. In conventional trials, interim data access is tightly restricted and governed by independent oversight, particularly when unblinded data could influence conduct. RTCT environments may alter this balance if emerging signals, including early trends in tumor response rates, biomarker changes, adverse event rates, recruitment patterns, subgroup accrual, or dropout, become visible before data are fully validated. These signals are especially unstable in early-phase trials, where small sample sizes increase the likelihood that observed patterns reflect random variation rather than true effects. Even without formal intervention, unvalidated signals may influence recruitment, clinical management, or sponsor decision-making, introducing selection bias, protocol drift, and analytic multiplicity that weaken controlled inference. Without explicit governance on data access, blinding, aggregation, and operational response, data visibility itself may function as an unmeasured intervention, complicating endpoint interpretation and threatening internal validity.

## Issue 4: Equity, infrastructure, and the risk of systematic exclusion

A critical and underexamined concern is the potential for RTCTs to exacerbate inequities in clinical research participation. RTCTs may be more readily implemented in academic medical centers with established research and digital infrastructure, yet these centers represent a minority of care settings and are disproportionately located in urban areas. Community hospitals account for approximately 85% of U.S. hospitals and serve most patients, but are less likely to support complex, data-intensive trial models, raising concerns that RTCT implementation may widen geographic and socioeconomic disparities in trial access and representativeness^8^. Real-time data capture and reporting require interoperable electronic health records, reliable connectivity, validated digital tools, and data governance capabilities that are not uniformly available across clinical settings, while current RTCT proposals provide limited clarity regarding how lower-resourced sites will be supported. Consequently, community hospitals, safety-net providers, and rural health systems may struggle to meet the operational demands of RTCT participation without substantial infrastructure and workforce investment. If participation becomes contingent on advanced digital capabilities, lower-resourced sites may become systematically underrepresented, limiting both access to trials and the generalizability of the evidence generated. Systematic underrepresentation of community, rural, and safety-net populations would not only reduce access to trials but could also limit the external validity and generalizability of evidence generated within RTCT ecosystems (Fig. 1).

## Solution: A governance framework for real-time clinical trials

The RTCT landscape remains nascent, and most clinical sites currently lack the infrastructure, validated pipelines, and workforce capacity that full participation would demand. However, these realities argue for building sound governance now, because frameworks designed in parallel with the technology they regulate are far more likely to shape it well than those retrofitted after scaling. That governance cannot be a static set of thresholds, rules, and policies, but must function as a dynamic operating system capable of keeping pace with evolving technology and operational demands, even as the three foundational pillars proposed here remain constant.

**Fig. 2:**
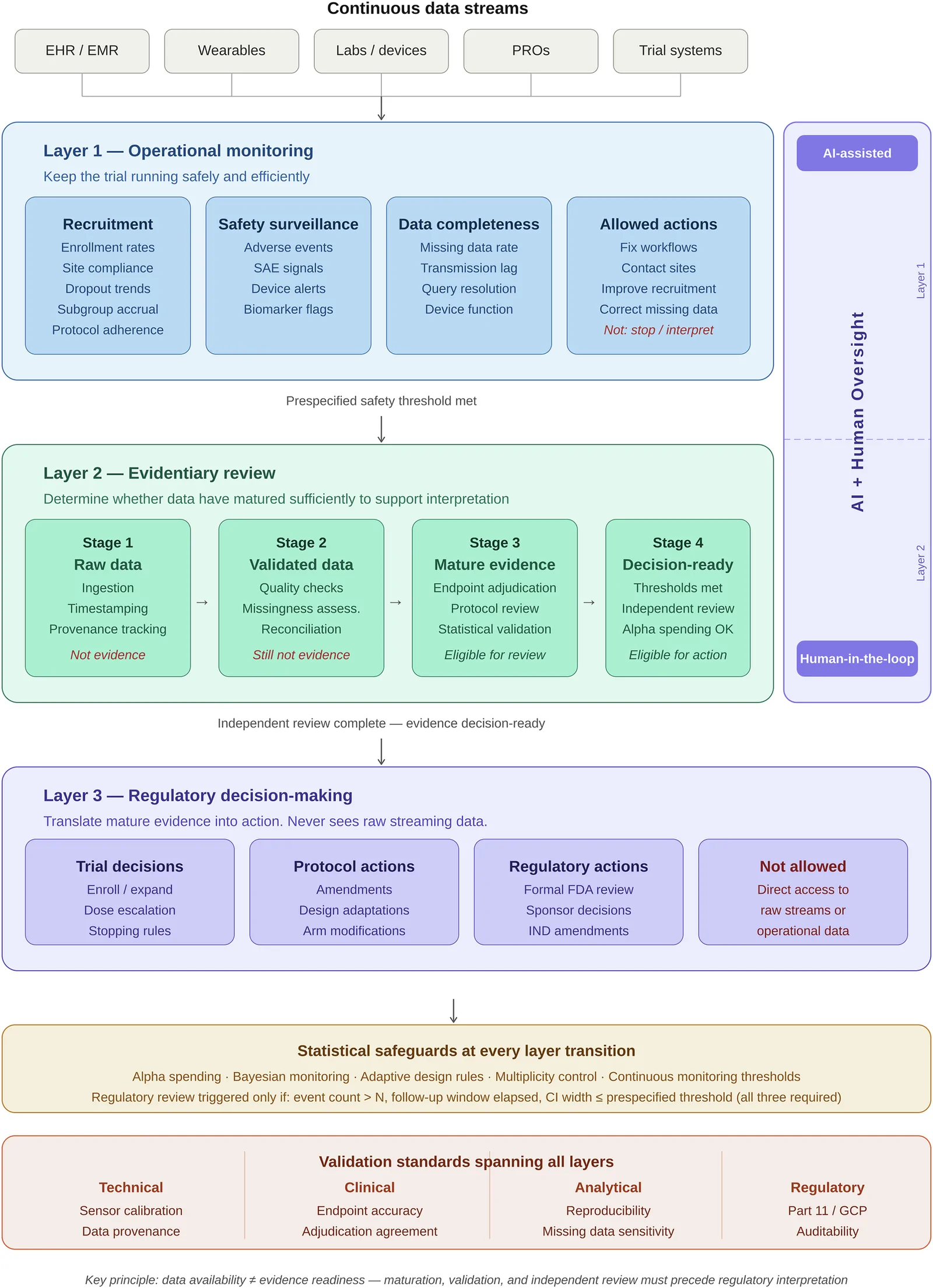
Separation-of-functions governance architecture for real-time clinical trials. Three governance layers regulate progression from continuous data streams to regulatory action: operational monitoring, evidentiary review, and regulatory decision-making. Evidentiary review applies a four-stage evidence maturation framework (raw data, validated data, mature evidence, and decision-ready evidence), while regulatory decisions are based only on decision-ready evidence. Prespecified thresholds, validation standards, statistical safeguards, and AI-assisted human-in-the-loop oversight govern all layer transitions. EHR electronic health record, SAE serious adverse event, PRO patient-reported outcome, GCP Good Clinical Practice, CI confidence interval.

The central risk of RTCTs is that continuous data visibility will blur the boundaries between monitoring, interpretation, and regulatory action collapsing three functions that must remain distinct if evidence is to retain its integrity. Separation of functions is therefore a core component of the proposed governance operating system, distinguishing real-time operational monitoring (Layer 1), evidentiary review (Layer 2), and regulatory decision-making (Layer 3), analogous to models in aviation safety, financial regulation, and Data Safety Monitoring Boards (Fig. 2). Layer 1 tracks recruitment, safety signals, protocol adherence, and data completeness using AI-assisted systems, restricted to operational corrections only and generating no evidentiary products, efficacy conclusions, or stopping recommendations, with each layer firewalled from unvalidated upstream data until prespecified evidentiary thresholds are met.

The core evidentiary risk of RTCTs is that continuously streaming data will be interpreted before it is stable or mature enough to support regulatory-grade conclusions. Addressing this requires robust data maturation standards as a core component of the governance operating system, operationalizing the fundamental distinction between data availability and evidence readiness. Layer 2 governs a four-stage maturation framework progressing from raw ingestion (Stage 1), quality assessment and reconciliation (Stage 2), endpoint adjudication and analytical confirmation (Stage 3), to decision-ready evidence eligible for regulatory action (Stage 4), with each transition requiring human-in-the-loop validation of reproducibility, robustness, sensitivity to missingness, auditability, and 21 CFR Part 11 compliance, alongside prespecified statistical safeguards that control the growing risk of false conclusions each time accumulating data are reviewed, and regulatory action triggered only when predefined criteria for data quality, follow-up duration, event accumulation, and statistical certainty are simultaneously satisfied^9–12^.

The risk that RTCTs may systematically exclude the sites that serve most patients makes infrastructure democratization the third and equally indispensable component of the governance operating system. Without interoperable electronic health records, modular cloud-based reporting, secure data pipelines, and centralized technical support, community hospitals, federally qualified health centers, safety-net providers, and rural systems cannot participate without extensive local digital infrastructure they do not have, and resource-rich academic centers will become the default RTCT participants, reproducing precisely the structural inequities the program should be designed to address^13^. Although RTCTs hold genuine promise, their success depends on whether the governance operating system, evidentiary maturation standards, and inclusive infrastructure proposed here are built with the same urgency as the data systems driving them because in continuously learning health systems, governance and innovation are not competing priorities but inseparable ones.

## Supplementary information

Transparent Peer Review file

## Data Availability

No data are available for this manuscript.
