# Supplementary material for: Governing accelerated evidence in real-time clinical trials
Source: Commun Med (Lond). 2026 Oct 28;6:566. doi: 10.1038/s43856-026-01909-8 (PMC13720012; doi:10.1038/s43856-026-01909-8)
Supplement: Supplementary file 1 [file 43856_2026_1909_MOESM1_ESM.pdf]

nature portfolio

Peer Review File

Governing Accelerated Evidence in Real-Time Clinical Trials

Corresponding Author: Professor Azizi Seixas

This file contains all reviewer reports in order by version, followed by all author rebuttals in order by version.

Version 0:

Reviewer comments:

Reviewer #1

(Remarks to the Author)
1. The author's manuscript describes potential issues associated with the FDA's real-time clinical trials program and proposes a novel solution. While this may be a worthy topic, insufficient detail is provided to make a convincing case to potential readers.
2. Earlier this year, the FDA announced a series of major steps to implement real-time clinical trials. FDA Commissioner Marty Makary, M.D. said the real-time clinical trial approach will allow FDA scientists to view safety signals and endpoints in real time as a trials progress. The agency's ultimate goal is stated as running real-time continuous trials across all phases of drug development.
3. In the present, the FDA is focused on safety signals and endpoints. How the agency will get to its ultimate goal of real-time continuous trials across multiple drug development phases was not specified. Hence, it is not known whether the issues raised in figure 1 will materialize in the future and whether the solution proposed in figure 2 will be applicable.

Reviewer #2

(Remarks to the Author)
This manuscript proposes a systematic way of thinking about governance of "real-time clinical trials", a concept proposed by recent FDA pronouncements. The logic is interesting and worthwhile. The stages and separation of function make sense. Some considerations:

1. The amount of work described, albeit very breifly, in this manuscript begs the question of whether this is a good idea after all. The average reader would benefit, at the least, from some description of how far we are from the steps outlined in the manuscript.

2. It seems very likely that as we move towards more rapid exchange of date, we'll learn a lot about how to do this. Some explicit acknowledgement that the basic framework is a starting point might be a good idea.

3. Most FDA experts think the proposal was more of a press event than a reality given the current state of technology-human interactions. Most therapies entering phase I (>90%) don't work and never make it to market. Among those that do make it to market, we have a real problem with figuring out which ones are most effective for which people and many of them require long-term followup to know. Wouldn't we do better to focus differently?

Version 1:

Reviewer comments:

Reviewer #1

(Remarks to the Author)
1. The changes are acceptable.
2. I have no additional comments.

Reviewer #2

(Remarks to the Author)
Thank you for responding to the critique.

Open Access This Peer Review File is licensed under a Creative Commons Attribution 4.0 International License, which permits use, sharing, adaptation, distribution and reproduction in any medium or format, as long as you give appropriate credit to the original author(s) and the source, provide a link to the Creative Commons license, and indicate if changes were made.
In cases where reviewers are anonymous, credit should be given to 'Anonymous Referee' and the source.
The images or other third party material in this Peer Review File are included in the article's Creative Commons license, unless indicated otherwise in a credit line to the material. If material is not included in the article's Creative Commons license and your intended use is not permitted by statutory regulation or exceeds the permitted use, you will need to obtain permission directly from the copyright holder.
To view a copy of this license, visit https://creativecommons.org/licenses/by/4.0/

Response to Reviewers

Manuscript: Governing Accelerated Evidence in Real-Time Clinical Trials
Journal: Communications Medicine
Manuscript Number: COMMSMED-26-1899-T
Date: August 2026

I thank the Editor and both Reviewers for their constructive feedback on this manuscript. I have addressed all comments. All changes are bolded and highlighted in the accompanying files: Tracked Manuscript and Clean Manuscript. See a detailed point-by-point response below:

Response to Reviewer #1 (Clinical Trials Expertise)

Reviewer #1, Comment 1–2: The author's manuscript describes potential issues with the FDA's real-time clinical trials program and proposes a novel solution. While this may be a worthy topic, insufficient detail is provided to make a convincing case to potential readers. The FDA Commissioner has stated the agency's ultimate goal is running real-time continuous trials across all phases of drug development.

Response: I thank Reviewer #1 for this critical but constructive framing. I agree that contextualizing the FDA's stated trajectory is essential to demonstrate why the governance challenges identified are not theoretical but immediate. The revised Introduction now explicitly notes that the FDA's program begins with current proof-of-concept safety and efficacy streaming (exemplified by the TRAVERSE and STREAM-SCLC initiatives) and that the agency's stated ambition is to extend real-time continuous trials across all phases of drug development. This trajectory makes the governance architecture, data maturation standards, and infrastructure requirements described here not speculative but preconditions for any scalable implementation. I further acknowledge, consistent with Reviewer #2's comment, that this framework is a starting point that will require iterative refinement as regulatory experience and technological capacity mature.

Reviewer #1, Comment 3: In the present, the FDA is focused on safety signals and endpoints. How the agency will get to its ultimate goal of real-time continuous trials across multiple drug development phases was not specified. Hence, it is not known whether the issues raised in Figure 1 will materialize in the future and whether the solution proposed in Figure 2 will be applicable.

Response: This is a well-taken point. I have added a passage to the Introduction that explicitly names the FDA's current scope (Phase I–II safety and efficacy streaming) and acknowledges that the governance challenges presented scale with the ambition of the program. Rather than treating the full RTCT vision as established, I now frame the paper as providing the governance architecture necessary to avoid foreseeable problems as the program expands. The two-sentence preface also signals this prospective framing. I also note that the proof-of-concept trials cited (TRAVERSE, STREAM-SCLC) are already operational, meaning that Questions 1 and 2 of Figure 1 (data velocity and GCP challenges) are not future risks but present ones.

Response to Reviewer #2 (Clinical Trials and FDA Policy Expertise)

Reviewer #2, Comment 1: The amount of work described, albeit very briefly, in this manuscript begs the question of whether this is a good idea after all. The average reader would benefit, at the least, from some description of how far we are from the steps outlined in the manuscript.

Response: I agree entirely that situating the proposed framework relative to current technological and operational readiness is essential for the reader. I have added a paragraph to the Solution section explicitly acknowledging that significant gaps exist between current technological-human systems and the demands of continuous, multi-phase real-time trials. I note that most clinical sites, including many academic centers, currently lack interoperable infrastructure, validated data pipelines, and the workforce capacity required for full RTCT participation. The framework is explicitly positioned as a prospective governance architecture and starting point not a description of existing practice that must evolve in response to implementation experience and regulatory learning.

Reviewer #2, Comment 2: Some explicit acknowledgement that the basic framework is a starting point might be a good idea.

Response: Adopted. The phrase 'starting point' now appears explicitly in the revised Introduction and in the Solution section. I state that the framework proposed here is explicitly offered as a starting point, recognizing that iterative learning, technological development, and regulatory experience will require refinement over time. This language directly addresses your suggestion and reflects my view of how governance frameworks in nascent fields necessarily evolve.

Reviewer #2, Comment 3: Most FDA experts think the proposal was more of a press event than a reality given the current state of technology-human interactions. Most therapies entering Phase I (>90%) don't work and never make it to market. Among those that do make it to market, I have a real problem with figuring out which ones are most effective for which people and many require long-term follow-up. Wouldn't we do better to focus differently?

Response: This is a substantive and important feedback. First, I now explicitly acknowledge in the Issue 1 section that more than 90% of Phase I therapies fail to reach market, which means that governance frameworks for RTCTs must be designed for the far more common scenario of null or inconclusive signals—not only for successful trials. Second, I note that many conditions requiring long-term follow-up (neurodegenerative diseases, cardiovascular outcomes, oncology endpoints) cannot rely on real-time data transmission as a substitute for adequate observation duration, and I raise this explicitly as a boundary condition for the framework's applicability. Third, the Reviewer's broader question whether the FDA's RTCT initiative represents substance or signaling is acknowledged in our introductory framing, where I now state that the framework is offered as a starting point that will need to be tested against implementation realities. I do not take a position on the FDA's institutional motivations, as that falls outside the scope of this Comment; however, I believe that providing a governance architecture for the scenario in which RTCTs do expand is a useful contribution to the field regardless of the timeline or ultimate scope of implementation.

I hope the revised manuscript and this point-by-point response address the reviewers' concerns fully. I remain available to provide any further clarifications requested by the Editor or Reviewers.

Sincerely,

Azizi A. Seixas
Department of Informatics and Health Data Science
Department of Psychiatry and Behavioral Sciences
University of Miami Miller School of Medicine, Miami, FL, USA
axs8514@miami.edu
